# Strategies for induction of HIV‐1 envelope‐reactive broadly neutralizing antibodies

**DOI:** 10.1002/jia2.25831

**Published:** 2021-11-21

**Authors:** Wilton B. Williams, Kevin Wiehe, Kevin O. Saunders, Barton F. Haynes

**Affiliations:** ^1^ Human Vaccine Institute Duke University School of Medicine Durham North Carolina USA; ^2^ Department of Surgery Duke University School of Medicine Durham North Carolina USA; ^3^ Department of Medicine Duke University School of Medicine Durham North Carolina USA; ^4^ Department of Immunology Duke University School of Medicine Durham North Carolina USA

**Keywords:** bnAb precursors, broadly neutralizing antibodies (bnAbs), Env immunogen design, HIV‐1 vaccine strategies, HIV‐1 vaccines, improbable mutations

## Abstract

**Introduction:**

A primary focus of HIV‐1 vaccine development is the activation of B cell receptors for naïve or precursor broadly neutralizing antibodies (bnAbs), followed by expansion and maturation of bnAb B cell lineage intermediates leading to highly affinity‐matured bnAbs. HIV‐1 envelope (Env) encodes epitopes for bnAbs of different specificities. Design of immunogens to induce bnAb precursors of different specificities and mature them into bnAb status is a goal for HIV‐1 vaccine development. We review vaccine strategies for bnAb lineages development and highlight the immunological barriers that these strategies must overcome to generate bnAbs.

**Methods:**

We provide perspectives based on published research articles and reviews.

**Discussion:**

The recent Antibody Mediated Protection (AMP) trial that tested the protective efficacy of one HIV‐1 Env bnAb specificity demonstrated that relatively high levels of long‐lasting serum titers of multiple specificities of bnAbs will be required for protection from HIV‐1 transmission. Current vaccine efforts for induction of bnAb lineages are focused on immunogens designed to expand naïve HIV‐1 bnAb precursor B cells following the recent success of vaccine‐induction of bnAb precursor B cells in macaques and humans. BnAb precursor B cells serve as templates for priming‐immunogen design. However, design of boosting immunogens for bnAb maturation requires knowledge of the optimal immunogen design and immunological environment for bnAb B cell lineage affinity maturation. BnAb lineages acquire rare genetic changes as mutations during B cell maturation. Moreover, the immunological environment that supports bnAb development during HIV‐1 infection is perturbed with an altered B cell repertoire and dysfunctional immunoregulatory controls, suggesting that in normal settings, bnAb development will be disfavoured. Thus, strategies for vaccine induction of bnAbs must circumvent immunological barriers for bnAb development that normally constrain bnAb B cell affinity maturation.

**Conclusions:**

A fully protective HIV‐1 vaccine needs to induce durable high titers of bnAbs that can be generated by a sequential set of Env immunogens for expansion and maturation of bnAb B cell lineages in a permitted immunological environment. Moreover, multiple specificities of bnAbs will be required to be sufficiently broad to prevent the escape of HIV‐1 strains during transmission.

## 1 INTRODUCTION

A goal of a highly effective HIV‐1 vaccine is to generate broadly neutralizing antibodies (bnAbs) targeting the envelope (Env) surface protein in order to prevent HIV‐1 infection — a global health priority. However, bnAbs have features of autoreactive antibodies, thus making them disfavoured for production by host immunity — as discussed in detail in previous reviews [1, 2, 3]. Additionally, HIV‐1 is a rapidly mutating RNA retrovirus that integrates into the host genome within ∼72 h after infection, and once integrated, forms a latent reservoir of infected cells that resists immune system elimination [4, 5, 6]. Thus, a successful HIV‐1 vaccine must have long‐lived, high levels of protective immunity that effects sterilizing immunity and completely prevents infection — a high bar that no other vaccine has had to reach.

**Figure 1:**
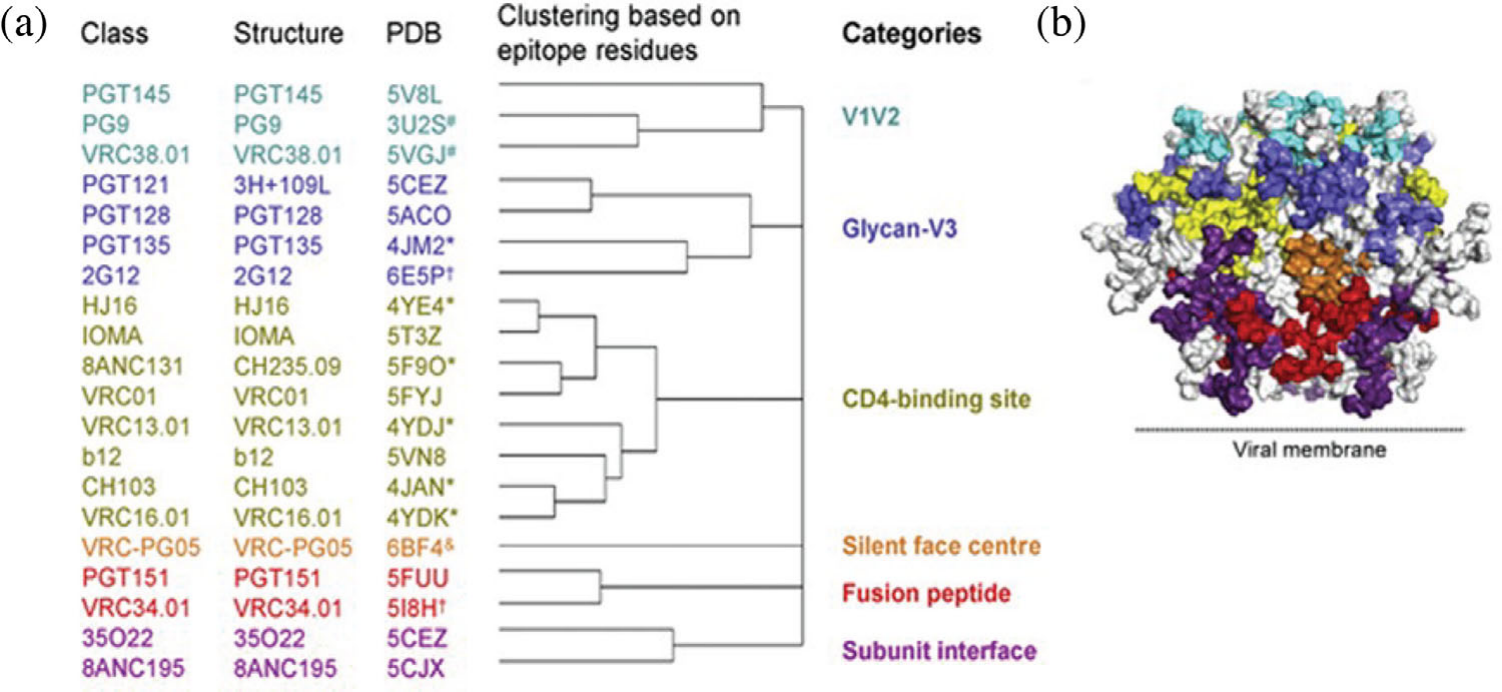
Classification of HIV‐1 Env‐reactive bnAbs. (A) Twenty classes of bnAbs that recognize the prefusion‐closed Env trimer have been segregated into six categories based on their Env residues of interaction. An additional class of bnAbs that target gp41 membrane proximal external region (MPER) (12) is not shown. All HIV‐1 Env‐antibody complex structures were assigned to classes; leftmost column lists the name of first reported antibody in each class. Each antibody class was categorized based on similarities in B cell ontogeny and mode of recognition. The protein data bank (PDB) IDs shown are for the structures of representative Env‐Ab complexes for Abs within each class. The PDBs were chosen based on resolution and degree to which Env in the structure resembled prefusion‐closed trimer as described (11). (*) indicates structures determined in deglycosylated gp120‐core context; (&) indicates structures determined in partially glycosylated gp120‐core context; (#) indicates structures determined with V1/V2 scaffold; (†) indicates a structure with high resolution peptide‐ or glycan‐antibody complex but lower resolution Env trimer‐antibody complex structure. (B) Prefusion‐closed Env trimer with molecular surface colored by categories defined in (A). This figure was adapted with permission from Chuang et al. (11).

Vaccine‐induced neutralizing antibodies have been effective against many viruses [7], and serum neutralizing antibodies have been shown to correlate with protection from autologous infection in animal models of HIV‐1 infection [8]. BnAbs target susceptible Env sites that are generally conserved across geographically diverse HIV‐1 strains and are thus required via vaccination to prevent the acquisition of infection [9, 10]. A recent structural survey of HIV‐1 Env bnAb targets by Chuang and colleagues described six bnAb epitopes on HIV‐1 Env gp120 (Figure 1) [11]. In addition, Caillat and colleagues summarized the structure and function of bnAbs targeting the membrane proximal external region (MPER) of Env gp41 near the gp41‐viral lipid bilayer interface [12]. Over 50% of the molecular mass of the HIV‐1 Env is comprised of glycans that mask neutralizing epitopes [13, 14, 15], and therefore, recognition of glycans is key to bnAb development [16, 17]. However, HIV‐1 Env has evolved to mimic glycosylated host proteins, resulting in minimal recognition of neutralizing epitopes by the immune system [18]. HIV‐1 Env also contains many variable loops that can be immunogenic [19], leading to diverting or “off‐target” antibodies that may out‐compete neutralizing antibodies [20, 21]. Thus, HIV‐1 Env composition also complicates the efforts for generating bnAbs.

Previous human HIV‐1 vaccine efficacy trials that studied first‐generation HIV‐1 Env vaccines did not generate bnAbs and showed either modest success [22, 23] or no protection [24, 25, 26]. Recent studies have implied that the frequency of candidate bnAb precursor B cells that possess the immunogenetics, functional and structural properties to mature into bnAb status is limiting [27, 28, 29]. Thus, an effective HIV‐1 vaccine will need immunogens to engage and mature rare precursor B cell lineages — concepts known as bnAb lineage vaccine design [30] and germline targeting [29, 31]. Using information from antibody‐virus co‐evolution for immunogen design is termed “lineage‐based” vaccine design [30]. Keys to this strategy include inference of a bnAb lineage unmutated common ancestor (UCA) B cell receptor (BCR) and design or usage of an optimal Env immunogen capable of expanding this pool of precursor B cells expressing the UCA BCR [30]. Combining structural and immunological information for certain classes of bnAbs, such as the CD4‐binding site (bs) antibodies of the VRC01 class, has led to an approach termed “germline targeting” [29, 31, 32]. Both concepts incorporate the use of immunogens that engage precursor B cells from which bnAbs affinity mature, but germline targeting begins with the estimation that critical bnAb precursor features are sufficiently common within and among different individuals to make them targetable by immunogens [10]. These vaccine concepts are being investigated in human clinical trials led by the HIV Vaccine Trials Network (HVTN).

During bnAb lineage maturation, bnAbs acquire genetic mutations at sites that are rarely targeted for somatic hypermutation, resulting in a low probability of their occurrence in germinal centres (GCs) during affinity maturation — these mutations were described as improbable mutations that impact antibody structure and affinity for antigen to improve antibody function [33, 34]. Improbable mutations have been shown to modulate neutralization breadth of the 8ANC131/CH235 class antibody lineages that target the CD4bs [33, 35, 36, 37], and the DH270 V3 glycan bnAb lineage [34, 36]. Additionally, the germline sequences of bnAbs encode paratope structural features that confer recognition of their conserved epitopes on Env. For example, the VH gene‐restricted CD4‐mimic bnAbs use specific immunogenetics for HCDR2‐mediated recognition of the CD4bs: VRC01 class of antibodies use VH1‐2*02 paired with light chain genes bearing CDR3s of five amino acids (aa) in length to avoid steric clashes with Env loop D and facilitate maturation to breadth [38, 39], whereas the 8ANC131/CH235 class of antibodies use VH1‐46 paired with various light chain genes of a range in CDR3 lengths [39]. BnAbs targeting gp41 MPER demonstrated heterogeneity in heavy and light chain gene pairs [12], but a hallmark of MPER bnAbs is a hydrophobic HCDR3 for effective epitope recognition in a two‐step process of interaction [40]. DH270 V3 glycan bnAb used a functional improbable mutation to displace the Env V1 loop and increase access to the bnAb epitope (N332 and GDIR motif) [34, 36]. These studies demonstrate that the most potent bnAbs are restricted in their immunogenetics, have improbable point mutations and/or have long (and sometimes hydrophobic) HCDR3s. Thus, successful HIV‐1 Env immunogens will need to select bnAb lineage B cells with restricted immunogenetics and improbable functional mutations that facilitate antibody maturation.

Here, we review strategies for vaccine induction of HIV‐1 Env bnAbs, which include multiple different approaches by different researchers. These strategies were informed by preclinical studies in mice and rhesus macaques, and some are currently being tested in humans. Moreover, we highlight immunological barriers that an effective HIV‐1 Env bnAb‐inducing vaccine must overcome.

## 2 METHODS

We referenced 131 research articles and reviews that were published between 2001 and 2021 in peer‐reviewed journals, and a press‐release in 2021. These references were accessed from PubMed between 10 May 2021 and 6 September 2021 when this review was written and revised. We referenced the initial research articles that report seminal discoveries or concepts discussed in this review. For well‐established concepts that are commonly discussed in the field of HIV‐1 vaccine development and perspectives, we reference review articles.

## 3 DISCUSSION

**Figure 2:**
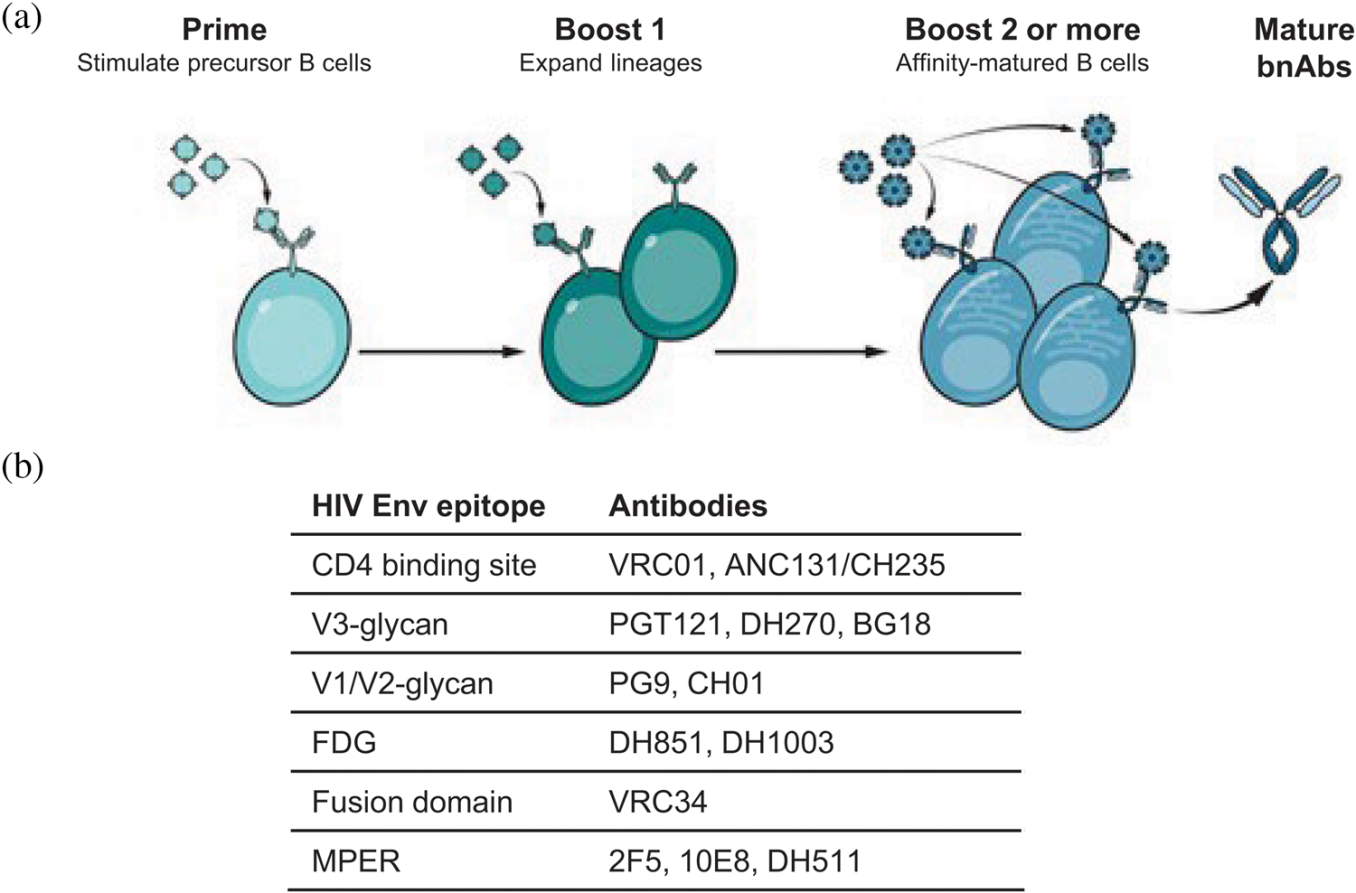
Strategies for bnAb induction via vaccination. (a) Sequential immunizations with a priming and boosting immunogens will expand B cell lineages from precursor to bnAb status. (b) Examples of antibodies that target different bnAb epitopes on HIV‐1 Env. The goal of a vaccine in the Duke CHAVD program is to induce different specificities of bnAb B cell lineages. This image was modified from [10].

Many HIV‐1 sexually transmitted infections are caused by a limited number of transmitted/founder (TF) viruses [41]. Antibody‐virus co‐evolution studies have demonstrated an “arms race” between the evolving viral quasi‐species and host neutralizing antibody responses such that TF viruses induce autologous neutralizing antibodies, but soon escape them, leading to additional neutralizing antibodies. In some individuals, virus Env proteins evolve such that over multiple virus mutation‐antibody neutralization cycles, eventually BCRs are selected with broad reactivity [30]. Thus, successful immunization regimens will likely require sequential immunizations to prime and then boost bnAb lineages with the necessary affinity maturation to recognize Env and potently neutralize HIV [10, 29, 30, 31] (Figure 2). This complexity of immunogen design is unprecedented, and is requiring small iterative phase 1 experimental medicine clinical studies with fewer participants in collaboration with the HVTN to work out feasible and logistically practical immunization regimens that can induce broad and durable serum bnAb activity [10].

### 3.1 Requirements for vaccine‐induced antibody protection from HIV‐1 acquisition

The AMP trials conducted by the HVTN and HIV Prevention Trials Networks (HPTN) studied at‐risk cisgender men and transgender persons in the Americas and Europe (HVTN 703/HPTN 081) and at‐risk women in sub‐Saharan Africa (HVTN 703/HPTN 081). Participants were randomized to receive infusion of the VRC01 bnAb every 8 weeks at doses of either 10 or 30 mg/kg, or placebo, over 20 months [42]. Overall, the trial did not prevent HIV‐1 infection, but it did answer two important questions for the HIV‐1 vaccine development effort [43]. First, passive immunization with VRC01 did protect against acquisition of HIV‐1, but only against viruses that were highly sensitive to the antibody. The level of neutralizing antibody found to be protective at the time of the HIV‐1 transmission was a relatively high serum ID80 titer of ∼1:250 [42], thus implying that high levels of protective antibodies will need to be present at the time of transmission for a bnAb‐inducing HIV‐1 vaccine to be effective. Secondly, the AMP trial showed that serum neutralization titer, as measured in a standardized pseudovirus assay, may predict protection, thereby providing an important analytic tool for future trials [43].

### 3.2 Strategies for induction of BNABS

Retrospective studies in people living with HIV‐1 who generate bnAbs have identified immunologic perturbations associated with bnAb induction [44, 45, 46, 47]. These studies indicated that a permissive environment for bnAb induction is associated with viral antigen persistence, constant immune activation, dysfunction of follicular regulatory CD4 T cells, reduced natural killer cell function and aberrant B cell repertoire that supports the development of autoreactive bnAbs. Thus, for bnAb induction via vaccination, immunogen strategies will need to overcome these immunological roadblocks [3, 48].

Animal models are key for testing immunogen strategies to elicit bnAbs. Knock‐in (KI) mouse models that express bnAb UCA VH and VL genes produce B cells bearing bnAb UCA BCRs serve as important tools to determine experimentally whether HIV‐1 Env priming or boosting immunogens can either expand bnAb precursors or as boosting immunogens select for affinity matured bnAb intermediate or mature antibodies [49, 50, 51]. Recently, the development of a simian‐human immunodeficiency virus (SHIV)‐infected macaque model of bnAb induction provides an important tool for prospective antibody‐Env coevolution studies and serves as a molecular guide to inform vaccine induction of bnAbs [52]. Using the SHIV model of bnAb induction in macaques, we can identify Envs as candidate immunogens for bnAb lineage development, and determine the permissible immunological environment for bnAb induction that may be recapitulated via vaccination regimens. In this regard, identifying adjuvants that support distinct immunological environments will be key to the success of an effective HIV‐1 vaccine.

BnAb lineage induction by activation and expansion of bnAb precursor B cells, followed by bnAb lineage maturation are key concepts for vaccine induction of bnAbs. We review strategies for bnAb lineage induction and maturation, including vaccine immunogens that are widely being studied for bnAb lineage induction and maturation. We also review vaccine delivery platforms, including more recently attractive platforms for HIV‐1 vaccines. For example, lipid nanoparticles are widely employed in vaccine efforts against multiple infectious diseases, including SARS‐CoV‐2 [53, 54, 55, 56], influenza [57], and respiratory syncytial viruses [57, 58, 59]. The Moderna [60] and PfizerBioNTech [61] SARS‐CoV‐2 vaccines used a modified messenger ribonucleic acid (mRNA) encoding the SARS‐CoV‐2 spike protein encapsulated in a lipid nanoparticle as a delivery platform. mRNA‐based vaccines have previously shown remarkable success in inducing protective immunity against ZIKA virus (ZIKV) in rhesus macaques [62]. Thus, lipid nanoparticle encapsulating mRNA is an attractive delivery platform for HIV‐1 vaccines [63, 64].

#### 3.2.1 BnAb lineage induction

To successfully target bnAb precursor B cells, a better understanding of the human naïve B cell repertoire that is capable of Env recognition and maturation into bnAbs is essential [65]. The characteristics of precursor B cells of CD4bs bnAb VRC01, for example, are well‐defined, thus facilitating effective repertoire analyses of VRC01 precursors in humans and mice models [27, 28, 66, 67, 68]. For the myriad of bnAb precursors without such well‐defined features, more studies are needed to interrogate the immunogenetics, function and structure of these precursor B cells in the naïve B cell repertoire using high affinity antigens designed to bind recombinant bnAb UCA antibodies. Table 1 lists examples of bnAbs with genetic and functional properties that define the selection criteria for candidate precursors of these lineages.

**Table 1:** Criteria for HIV‐1 Env bnAb precursors

BnAb specificity	CD4‐binding site	V1V2	Env V3 glycan		MPER
BnAb IDs	CH103, CH235, VRC01	CH01	DH270	2G12, DH851‐like	2F5, 4E10, 10E8, DH511
Genetics	[CH103] V_H_4‐59 + V_L_3‐1; [CH235] V_H_1‐46 (W50/ N58/R71); [VRC01] V_H_1‐2 + 5‐aa LCDR3 (V_K_1‐33, V_K_3‐20, V_K_3‐15, V_L_2‐14)	Long anionic HCDR3; Tyrosine‐rich HCDR3; D‐D fusion	Long CDRs	Short HDR3; Hydrophobic residues in dimer interface	Hydrophobic HCDR3; Lipid reactivity; polyreactive
Binding	UCA‐reactive SOSIP^a^, ^b^; eOD‐GT8‐reactive^c^	UCA‐reactive SOSIP^d^	UCA‐reactive SOSIP^e^	Man_9_‐V3 reactive^f^	MPER peptide‐reactive^g^
	CD4bs‐KO sensitive	N160‐dependent	GDIR‐dependent; N332‐dependent	Glycan‐dependent	D664A/W672A‐sensitive
Neutralization	[CH103] CH505.w4.3, CH505TF.gly4; [CH235] CH505 M5_G458Y [VRC01] 426c.TM4/ GnTi‐	WITO, Q23, ZM233, T250‐4	CH848 10.17 DT; JRFL ΔV1 glycans	Kifunensine‐treated HIV‐1 isolates	Neutralization of isolates in TZM‐bl/FcgR1 assay

BnAb specificities that inform sort strategies for precursor B cell isolation.

^a^ [CH103] CH505 M11 SOSIPv4.1: WT versus S364K_T455E_G459E (CD4bs KO mutant).

^b^ [CH235] CH505 M5 SOSIPv4.1_G458Y/GnTi‐: WT versus N280D (CD4bs KO mutant) [35, 36].

^c^ [VRC01] eOD‐GT8: WT versus N279KD368R (CD4bs KO mutant) [27, 38, 68].

^d^ T250‐4 SOSIPv4.1: WT versus N160 (V2 apex KO mutant).

^e^ CH848 10.17 DS.SOSIP_N133DN138T: WT versus N332T (V3‐glycan KO mutant) [36].

^f^ Man_9_‐V3 glycopeptide: WT versus Aglycone V3 peptide (Env glycan KO mutant) [69].

^g^ MPER peptide: WT versus D664AW672A (MPER KO mutant).

**Figure 3:**
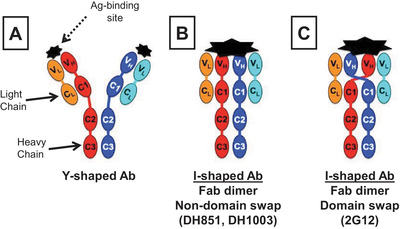
Schematic of antibody (Ab) structures. (a) A canonical Y‐shaped Ab has two independent antigen‐binding sites. (b) Fab dimerized glycan‐reactive (FDG) Ab with VH‐VH dimerized I‐shaped that has two Fabs acting as a single unit. (c) I‐shaped, domain‐swapped 2G12 bnAb with an additional binding site formed at the domain‐swap interface. Fab‐dimerized Abs have a large paratope for Env glycan recognition.

Understanding the population size of precursor B cells will identify attractive targets for vaccination and provides insights into the challenges for inducing bnAb lineages of different specificities. Precursors for bnAbs targeting the Env peptide backbone in conjunction with glycans are rare, approximately 1 in 54 million B cells [29], whereas candidate precursors for bnAbs targeting only Env glycans, termed Fab‐dimerized glycan‐reactive (FDG) antibodies, are more abundant at 1 in 340,000 B cells [69]. FDG antibodies use a unique VH‐VH Fab dimerized conformation to target Env glycans in contrast to the VH domain‐swapped conformation used by Env glycan bnAb 2G12 [70] (Figure 3), and we also found FDG B cells with an IgM+IgD+CD27+ marginal zone B cell phenotype [71], suggesting that FDG antibodies originated from the pool of glycan‐reactive natural antibodies [72]. FDG precursors were also reactive with yeast glycans, suggesting that high mannose‐bearing environmental antigens may prime these B cells, thus increasing their frequencies in the B cell repertoire to respond to glycosylated pathogens. For FDG B cell lineage development, we have identified high mannose‐bearing glycopeptide (Man_9_‐V3) that expanded FDG B cells in macaques and as a bait isolated candidate FDG precursor B cells from HIV‐1 naïve individuals. Man_9_‐V3 Star polymer composed of a 30‐mer array of Man_9_‐V3 glycopeptide [73] is a candidate immunogen for eliciting FDG antibodies in humans; a clinical trial is under development with NIAID Division of AIDS support.

#### 3.2.2 BnAb lineage maturation

GC reactions with persistent, high levels of CD4 follicular‐helper T cell (T_FH_) are required for efficient bnAb B cell affinity maturation [74, 75, 76, 77, 78]. In GC reactions, B cells can differentiate into long‐lived plasma cells that reside in bone marrow where they secrete and sustain serum antibodies as well as differentiate into memory B cells that disperse and reside both in secondary lymphoid tissues and bone marrow [79, 80]. Memory B cells can become reactivated by secondary antigenic encounters leading to sequential rounds of GC maturation [80, 81]. Studies to delineate the factors governing B cell fates have provided insights into the role of antigen valencies and affinities for recruiting bnAb precursor B cells into GC reactions [82, 83], but careful consideration is needed to choose immunogens with the appropriate structure, valency and affinity for prime and boosting immunogens to select bnAb B cell lineage members via vaccination [84]. A hallmark of bnAb lineage development is the acquisition of functional improbable mutations to be selected in GCs [33, 34, 35, 85], thus highlighting the need for innovative immunogen design to overcome genetic constraints to bnAb development by selecting functional mutations in bnAb lineages of multiple specificities.

Currently, HIV‐1 Env immunogens with varying affinities for bnAb UCAs are being tested in preclinical animal models and human clinical trials for their ability to elicit bnAb B cell lineages via vaccination [28, 36, 66]. These studies are investigating immunogens capable of priming bnAb precursor B cells and will then evaluate the binding characteristics of these expanded precursors to additional immunogens to identify candidate vaccine boosts. Most bnAb precursors are very rare and a concept is emerging that to expand bnAb precursors, vaccine regimens will need to prime with very high affinity immunogens [28, 36, 82, 83]. However, in studies in mice, it has been suggested that high affinity antigens select for B cells that differentiate into short‐lived plasma cells and leave the GC [86]. Thus, for boosting bnAb B cell lineages, tuning the affinity down to a lower level to keep the GC reaction going to favour affinity maturation and selection of BCRs with functional mutations may be required for achieving heterologous neutralization breadth [30, 36].

#### 3.2.3 Immunization strategies with monomeric Env gp120

The first antibody response to HIV‐1 Env in acute HIV‐1 infection is to gp41 that cross‐reacts with the gut microbiome [87, 88, 89]. Similarly, immunization with HIV‐1 Env in a DNA prime, rAd5 boost regimen in the HVTN 505 trial induced primarily gp41‐reactive antibodies that cross‐reacted with gut microbiome antigens [90]. The gp41‐microbiota cross‐reactive antibodies were non‐neutralizing and did not mediate Fc‐dependent anti‐viral functions, thus implicating a role for monomeric Env gp120 immunogens to circumvent a dominant gp41‐microbiota cross‐reactive B cell responses in vaccination [91]. It has also been hypothesized that immunization of neonates will “imprint” the B cell repertoire such that recognition of otherwise subdominant HIV‐1 Env bnAb epitopes will be promoted [92, 93]. To address the question whether a gp120 immunogen can prime bnAb precursor B cells in humans, CH505 TF gp120 is being tested in human adults (HVTN115, NCT03220724) and infants (HVTN135, NCT04607408).

We previously isolated a CD4bs antibody of the HCDR3‐dominated antibody type [39], referred to as CH103, from an individual chronically living with HIV‐1 (CH505) [94]. CH505 TF gp120 induced neutralizing CD4bs antibodies in macaques and clonally expanded CH103 UCA B cells in CH103UCA VH+VL KI mice [95]. Moreover, neonatal macaques and infants are capable of mounting B cell responses against HIV‐1 immunogens, including Env gp120 monomers [96, 97]. Thus, studying CH505 TF gp120 in human adults (HVTN115, NCT03220724) and infants (HVTN135, NCT04607408) will facilitate evaluation of the B cell repertoires that may support bnAb induction in both human adults and infants. If CH505 TF gp120 elicits bnAb precursor B cells in infants, then these data will provide insights into strategies for bnAb induction in children prior to sexual debut and provide supporting evidence for early life HIV‐1 immunization [98].

A modified gp120 protein designed to engage VRC01 germline and intermediate antibodies referred to as 426C core has shown promise in expanding VRC01 precursor B cells in mice when combined with other immunogens [99, 100, 101]. Thus, 426C core is another candidate gp120 immunogen that will be tested in human clinical trials for inducing or maturing CD4bs bnAb lineages.

#### 3.2.4 Immunization strategies with Env trimers

A current hypothesis in the HIV‐1 vaccine design field is that mimicking the structure of virion‐associated Env trimers with recombinant vaccine immunogens will be required for inducing neutralizing antibodies. The rationale behind this hypothesis is that antibodies raised to recognize non‐native conformations of Env will lack the ability to bind to fusion‐competent native Env on viruses. Recombinant SOSIP trimers were designed as near native‐like Env trimers that mainly display bnAb epitopes and have higher affinity for bnAbs than monomeric proteins [102, 103]. However, these trimers have a propensity for generating autologous tier 2 neutralizing and gp41 trimer‐base binding antibodies in animal models [104, 105, 106]. In addition, SOSIP trimer‐induced autologous tier 2 neutralizing antibodies can target glycan holes that are vulnerable regions on the trimer that lack potential N‐linked glycans [107]. Subsequent reports have shown that glycan hole‐targeted antibodies may be difficult to mature to breadth due to strain‐specific amino acid differences and the presence of glycans within such epitopes on other HIV‐1 isolates [108, 109]. Since autologous tier 2 serum neutralizing antibodies elicited by BG505 SOSIP trimer conferred protection to an autologous BG505 SHIV challenge in macaques [8], SOSIP trimers remain under consideration as candidate immunogens in an HIV‐1 vaccine, potentially for use as late boosts.

Previously isolated bnAbs have also been used to redesign SOSIP trimers for improved affinities as the next generation of immunogens. For example, BG18 germline and BG18‐like precursor antibodies were used to design and select the high affinity Envs N332‐GT2 and N332‐GT5 [29]. Structural analysis revealed that germline and mature BG18 antibodies demonstrated similar modes of binding to N332‐GT2 trimer [29, 110]. As an immunogen, N332‐GT2 trimer expanded BG18 precursor B cell lineages in BG18 germline‐VH KI mice, and as a bait isolated candidate BG18‐like human precursor antibodies in humans that shared the same HCDR3 length, D gene, D gene reading frame, D gene position within HCDR3 and JH gene with BG18 [29]. The design of N332‐GT2 was based on modifications to BG505 MUT11B SOSIP [111], which was unsuccessful at isolating PGT121‐like bnAb precursors [29]. Moreover, modified forms of the BG505 Mut11B SOSIP that were generated by removal of a V1 glycan at position 156 (RC1) in addition to introducing potential N‐linked glycan sites at gp120 positions 230, 241, 289 and 344 (RC4‐fill) generated serum antibodies and blood‐derived B cells with V3 glycan phenotypes, in contrast to BG505 Mut11B trimer [112].

Immunodominant B cell responses elicited by Env oligomers, including SOSIP trimers, are less desirable than bnAbs [113, 114], thus understanding how to avoid triggering these cells is important for vaccine induction of bnAb B cells. HIV‐1 BG505 SOSIP trimer (HVTN137, NCT04177355) and CH505TF SOSIP trimer (HVTN300, NCT04915768) are being studied in humans; the results of these trials will provide insights into the B cell responses to a near native‐like trimer, including antibody responses to bnAb versus non‐bnAb epitopes, thereby informing future Env immunogen design.

#### 3.2.5 Immunization strategies with multimeric forms of Env oligomers

Multimeric forms of HIV‐1 Env immunogens, including SOSIP trimers, are attractive vaccine candidates for bnAb induction [36, 82, 83, 115]. We and others have shown via antigenic and immunogenicity studies that nanoparticles were desirable for HIV‐1 Env bnAb induction [36, 57, 116, 117, 118]. The nanoparticle platform may be more advantageous for uptake by dendritic cells [83, 115, 119, 120], avid BCR engagement and increased antigen presentation that contributes to both enhanced B and T cell responses [64]. Improbable mutations that modulate bnAb lineage development require strong antigenic selection [33, 34, 35, 85], which may more likely occur with increased antigen presentation conferred by nanoparticle immunogens. Thus, bnAb lineage‐inducing HIV‐1 vaccine strategies under development by our group use nanoparticles that may be delivered via different platforms.

DH270, a V3 glycan bnAb B cell lineage derived from an individual living with HIV‐1 (CH848), provides a blueprint for vaccine induction of a V3 glycan bnAb lineage [34]. Co‐evolution studies of antibody reactivities and virus sequences from an individual living with HIV‐1 (CH848) identified potential Env variants with increasing affinities to the unmutated and affinity‐matured DH270 lineage antibodies [34, 36]. Importantly, a natural variant of the CH848 TF virus termed CH848 D0949.10.17 was identified that bound to lowly somatically mutated DH270 lineage antibodies. CH848 D0949.10.17 was engineered to remove the N133 and N138 V1 glycans, which enabled nanomolar affinity for DH270 UCA. This immunogen, termed V3G‐CH848 Pr‐NP1, has been investigated as a candidate immunogen to target the naïve BCRs capable of initiating a DH270‐like V3 glycan bnAb B cell lineage [36]. In DH270 UCA KI mice, V3G‐CH848 Pr‐NP1 formulated as a multimeric nanoparticle protein, indeed, expanded DH270 bnAb precursors and selected for critical antibody heavy and light chain improbable mutations required for heterologous neutralization [11]. The next steps in this mutation‐guided, B lineage vaccine design approach are to optimize Env immunogens of appropriate affinities for the bnAb lineage members induced, in order to continue to select for additional functional mutations needed for heterologous virus neutralization breadth — proof of concept for the strategy of targeting naïve V3‐glcyan B cells and bnAb lineage‐based vaccine design [30] for DH270 V3‐glycan bnAb. Similar success in inducing V3‐glycan bnAb precursors has been reported by others in V3 glycan bnAb early lineage KI mouse models [29, 112, 121] and in macaques [112].

**Figure 4:**
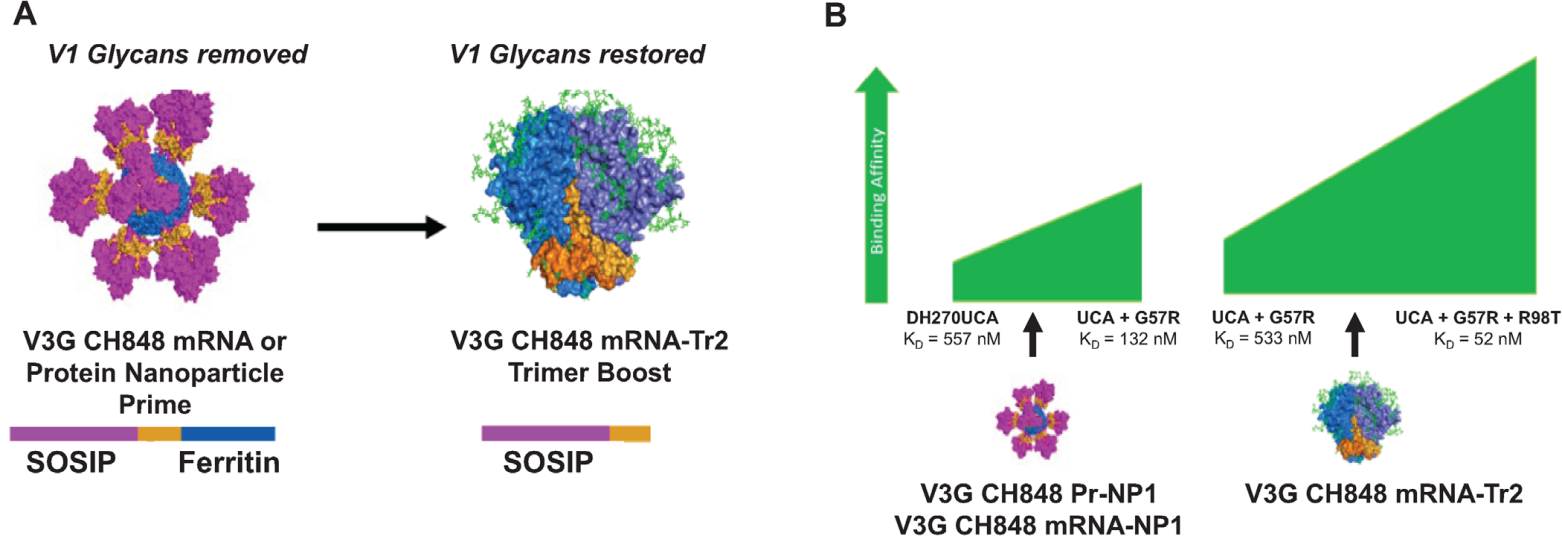
Lineage‐based vaccine strategy for inducing V3‐glycan bnAbs. (a) Candidate germline targeting priming and boosting immunogens to elicit DH270 V3 glycan bnAb B cell lineages. The priming immunogen will bear Envs with V1 glycans removed, whereas the boosting immunogen will express Envs with the V1 glycans restored. These vaccine immunogens will be formulated as either mRNA in lipid nanoparticles (NP) or as a protein (Pr) followed by a stabilized trimer (Tr) encoded by an mRNA. (b) Priming and boosting immunogens in panel a were selected based on an affinity gradient for recognition of the UCA and mature DH270 bnAb. Immunogen design included modifications of glycans that was informed by structural and functional studies of DH270 UCA and mature bnAb. The following strategy will be studied in humans in collaboration with the HVTN: V3G CH848 Pr‐NP1 + mRNA‐Tr2.

Lipid nanoparticle encapsulating mRNA that encode HIV‐1 Env has shown promise for eliciting neutralizing antibodies in animal models, some of which were durable responses [102, 103] likely due to the intrinsic adjuvant effects provided by the lipid nanoparticles [122]. It remains unknown if the mRNA lipid nanoparticle provides an advantage over conventional adjuvants to generate durable HIV‐1 Env antibody responses as observed for 3M‐052, a TLR‐7/8 agonist [123] or as cationic LNPs for protein immunogens [122] — a focus of future studies. The recent success of the mRNA‐based COVID19 vaccines has made this vaccine delivery platform attractive for HIV‐1 vaccines [63, 124, 125]. Moreover, we recently showed that lipid nanoparticle encapsulating mRNA can encode nanoparticles bearing HIV‐1 SOSIP trimers that initiate bnAb precursor B cell lineage expansion in bnAb precursor VH + VL KI mice [124]. We have proposed a strategy for DH270‐lineage development using either mRNA in lipid nanoparticles (V3G CH848 mRNA‐NP1) or protein (V3G CH848 Pr‐NP1) as the priming immunogen followed by a stabilized trimer encoded by an mRNA (V3G CH848 mRNA‐Tr2) as a boost (Figure 4). The SOSIP‐ferritin nanoparticle (V3G CH848‐NP) bound the UCA of DH270 V3 glycan bnAb (K_D_ = 557 nM) and had improved affinity for the DH270 UCA bearing the improbable G57R VH improbable mutation (K_D_ = 132 nM) [36], suggesting that V3G CH848‐NP immunogen may select for improbable mutations in DH270 lineage intermediate bnAb B cells and confer bnAb lineage maturation. Similarly, the SOSIP trimer (V3G CH848‐Tr) alone bound DH270 UCA (K_D_ = 533 nM) and had improved affinities for the DH270 UCA with the G57R VH improbable mutation (K_D_ = 52 nM). The V3G CH848 priming Env will have V1 glycans deleted and the trimer boost will have the V1 glycans restored, since the DH270 UCA bound with better affinities to V1 glycan‐deleted Envs, whereas the DH270 lineage intermediate antibodies and bnAbs learned to accommodate the V1 glycans while accessing the V3 glycan bnAb site using the G57R VH improbable mutation [36]. Both the UCA and mature DH270 had the same angle of approach for epitope recognition [36], thus indicating that structural analysis plays a key role in identifying candidate bnAb precursors. We will use a similar concept to find candidate prime and boost immunogens for bnAb lineages of different specificities. In this regard, we will initially test bnAb UCAs against Envs for measurable affinities, then identify boosting Envs that can bind to the UCA bearing improbable mutations with higher affinity than the UCA alone.

Other candidate multimeric immunogens have also been described for induction of bnAb lineages of different specificities. eOD‐GT8 was identified via a structure‐based immunogen design strategy as a high affinity germline‐targeting antigen for binding VRC01 CD4bs precursor antibodies [66]. Compared to lower affinity versions of eOD antigens, eOD‐GT8 was shown to be more effective at isolating VRC01 precursor B cells in HIV‐1 naïve humans and has been identified as a priming immunogen for VRC01 precursor B cells in humans [27]. Vaccine regimens for guiding VRC01 precursors into bnAb status must overcome nucleotide insertions and deletions (indels) in the antibody variable regions that occur during the maturation of the natural VRC01 lineage [31, 82, 99, 126]. The fusion peptide domain, a key element in the process of viral entry into host cells, has been recently identified as a vaccine‐inducing bnAb target in the Env gp41 subunit [127]. Strikingly, an Env fusion‐domain multimer generated via an epitope‐focusing immunogen design strategy elicited broadly neutralizing fusion‐peptide targeted antibodies that demonstrated 31% breadth in immunized mice [128] and 59% breadth in immunized macaques [129]. Thus, vaccine strategies containing fusion peptide immunogen are of interest for testing in human clinical trials [130]. However, additional boosts to eOD‐GT8 and fusion peptide priming immunogens must then be designed to continue to select for affinity‐matured antibodies with functional improbable mutations that confer increasing levels of neutralization breadth [29, 33].

### 3.3 Evaluation of BNAB B cell lineages in HIV‐1 vaccine clinical trials

Knowing the optimal fold change increase of vaccine‐induced bnAb precursors relative to baseline for subsequent bnAb induction is key to evaluating the success of vaccine strategies being tested in phase I human clinical trials to induce bnAb precursor B cells. Establishing such a criterion for bnAb precursors may be achieved by interrogating the immunogenetics, function and structure of bnAb precursor B cells in blood and other compartments where feasible (Table 1) [27, 66]. In addition to immunogenicity, phase I clinical trials evaluate reactogenicity and adverse events in vaccine trial participants to determine the safety of vaccine regimens (see protocols using ClinicalTrials.gov identifier).

HVTN115 (NCT03220724), HVTN300 (NCT04915768) and HVTN133 (NCT03934541) studied bnAb‐lineage vaccine design [30] in humans. CH505 TF Env gp120 with a low binding affinity for the CH103 UCA (K_D_ = 550 nM) is being studied in HVTN115 (NCT03220724) to determine if repetitive boosting with low affinity gp120 in GLA‐SE adjuvant can induce CH103 precursor B cells. For comparison, HVTN300 (NCT04915768) will study repetitive boosting of GLA‐SE adjuvanted, stabilized CH505 TF SOSIP trimers that have a higher binding affinity to CH103 UCA (K_D_ = 171 nM) than the CH505 TF gp120. HVTN115 (NCT03220724) is also studying sequential CH505 gp120s to test the hypothesis that CH505 TF gp120 can prime CH103 precursor B cells and sequential immunizations with natural variants of CH505 TF will mature these B cells towards breadth, in a manner that recapitulates viral Env and CH103 antibody co‐evolution in an individual living with HIV‐1 [94]. For immune monitoring of HVTN115 (NCT03220724) and HVTN300 (NCT04915768), serum antibodies and the B cell repertoire are being probed for antibodies that bind to CD4bs bnAb epitopes [94, 95].

Gp41 MPER liposome was studied in HVTN133 (NCT03934541) as a promising immunogen for eliciting MPER bnAb precursor B cells given that a first‐generation gp41 MPER peptide‐liposome‐containing vaccine regimen expanded 2F5‐like B cell precursors and intermediate antibodies in 2F5 bnAb precursor KI mice and rhesus macaques [131]. In HVTN133 (NCT03934541), vaccine trial participants received repetitive doses of gp41 MPER liposome adjuvanted in alum. For immune monitoring of HVTN133 (NCT03934541), serum antibodies and the B cell repertoire are being probed for antibodies that bind to MPER bnAb epitopes [132].

Additionally, eOD‐GT8 60mer was tested in healthy volunteers (IAVI G001) as a VRC01 germline‐targeting immunogen and expanded B cells with characteristics of VRC01 bnAb precursors (Table 1) in 97% of participants who received the vaccine [133]. This study demonstrated in humans that vaccination with germline‐targeting immunogens can achieve success engaging and expanding bnAb precursor B cells of interest in the B cell repertoire. As with all trials that test priming immunogens to elicit bnAb precursor B cells, the next step is to identify the boosting immunogen(s) to mature the B cells to bnAb status.

## 4 CONCLUSIONS

**Figure 5:**
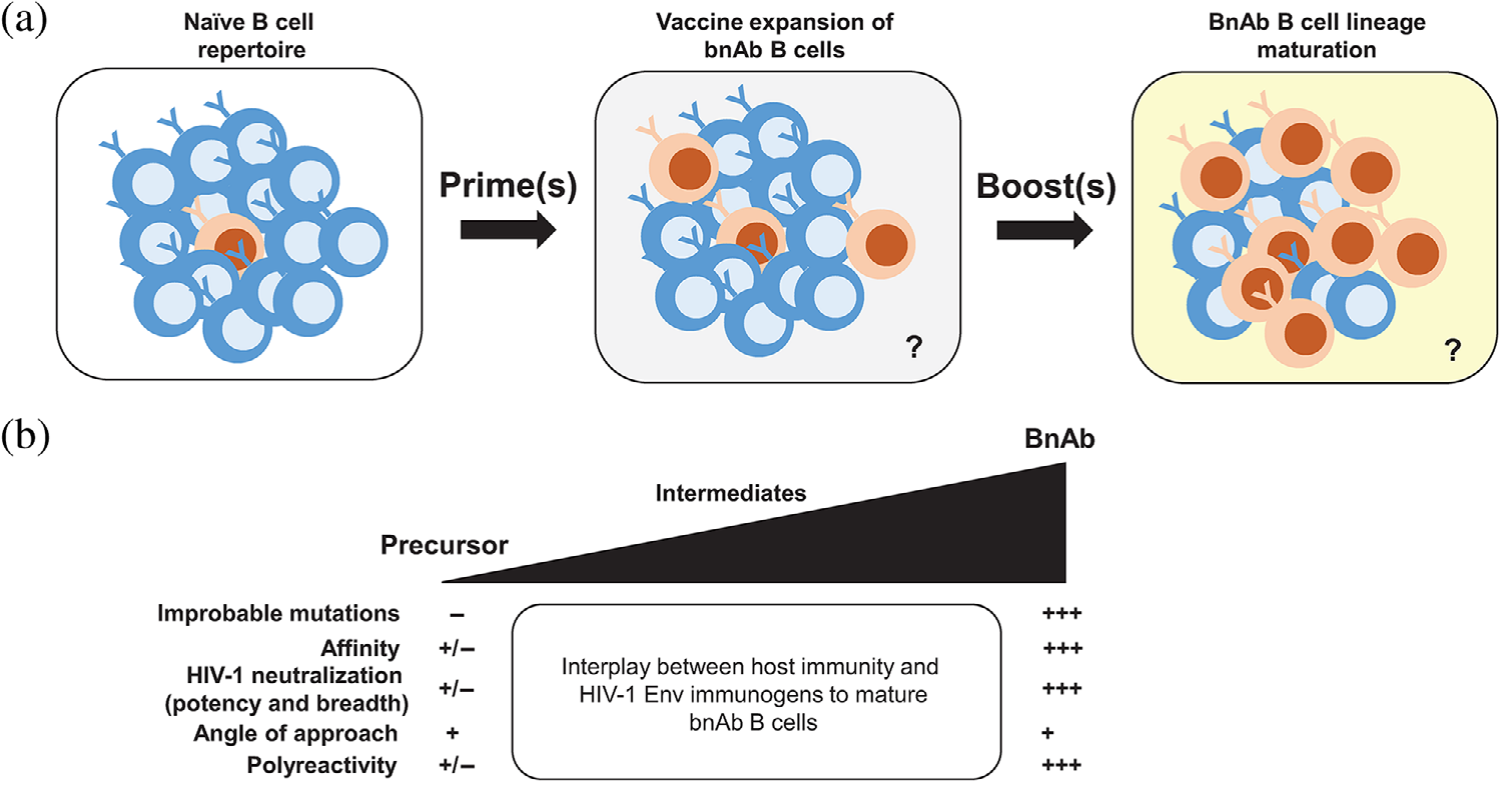
Vaccine engagement and maturation of bnAb precursor B cells in a permissive immunological environment. (a) Expansion of the pool of bnAb precursor B cells in the naïve repertoire following immunizations. In this review, we have discussed various strategies for bnAb lineage expansion that includes priming and boosting immunogens. BnAb lineage expansion in the cartoon is represented by an increase in the frequency of a bnAb B cell lineage (shown in orange) following sequential immunizations (prime and boost). BnAb lineage development will occur in a permissive immunological environment that is not fully established (denoted by question marks). (b) Properties of antibodies during maturation from precursors to intermediate and ultimately bnAb status. Changes in profiles are reflected by +++ symbols over +/– for baseline responses as precursor antibodies.

A successful bnAb‐inducing vaccine strategy will facilitate an interplay between host immunity and HIV‐1 Env immunogens to mature bnAb B cells (Figure 5). There are multiple pieces in the puzzle for solving an HIV‐1 vaccine to elicit bnAbs: understanding the target (i.e. the naïve B cell repertoire), immunogen design to engage bnAb precursor B cells and select mature B cell lineage members with functional improbable mutations, and vaccine delivery platforms and adjuvants to promote durable antibody responses. Therefore, making an HIV‐1 vaccine to induce globally effective, long lasting and safe bnAbs is a daunting task. Global communication and collaboration is essential to complete this difficult task. To accelerate vaccine development, the Collaborative HIV‐1 Immunogen Project (CHIP) has been formed, and comprised of the participants of the Duke and Scripps CHAVD, the NIAID Vaccine Research Center, the HVTN, the BMGF, International AIDS Vaccine Initiative and others funded around the world working on HIV‐1 vaccines [10]. The goal of CHIP is to promote communication and collaborations such that a successful HIV‐1 vaccine can be developed as soon as possible.

## COMPETING INTERESTS

BFH, KW, and KOS have patents submitted on select immunogens and concepts described in this review.

## AUTHORS’ CONTRIBUTIONS

WBW and BFH drafted the outline and wrote the review. KW and KOS edited the review.

## Data Availability

All the data reference have been published or will be shared in future publications.
